# Practical nomogram based on comprehensive CT texture analysis to preoperatively predict peritoneal occult metastasis of gastric cancer patients

**DOI:** 10.3389/fonc.2022.882584

**Published:** 2022-12-01

**Authors:** Shuxiang Chen, Huijuan Zhang, Hong Wei, Yongxiu Tong, Xiaofang Chen

**Affiliations:** ^1^ Department of Radiology, Provincial Clinical College of Fujian Medical University, Fujian Province Hospital, Fuzhou, China; ^2^ Department of Cadre Health Care Office, Provincial Clinical College of Fujian Medical University, Fujian Province Hospital, Fuzhou, China

**Keywords:** gastric cancer, peritoneal metastasis, nomogram, computed tomography, X-ray, texture analysis

## Abstract

**Objectives:**

This study aims to evaluate whether a nomogram based on comprehensive CT texture analysis of primary tumor and peritoneotome combined with conventional CT signs can preoperatively predict peritoneal occult metastasis in gastric cancer patients.

**Methods:**

A total of 1,251 patients with gastric cancer (GC) were retrospectively analyzed in Fujian Province Hospital between 2008 and 2020. Patients from the occult peritoneal metastasis (PM) group were initially diagnosed as PM-negative on CT and later confirmed as PM-positive through laparoscopy or surgery. The group without PM was randomly sampled from patients without PM. The preoperative CT signs and texture features and clinical characteristics of patients were retrospectively analyzed. Hazard factors of occult PM were identified by univariate analysis and multivariate logistic regression analysis, which were intended for creating prediction models. A nomogram was established based on the model with the highest predictive efficacy and clinical application value.

**Results:**

A total of 31 patients with occult PM and 165 patients without PM were enrolled in this study. The maximum size, thickness, enhancement, serous involvement of primary GC tumor and ascites on CT, and texture features such as inhomogeneity of the primary tumor, standard deviation, and inhomogeneity of the peritoneum were determined as independent predictors that could be jointly applied to predict occult PM. We separately constructed five forecast models using CT signs, primary tumor texture, peritoneum texture, primary tumor texture + peritoneum texture, and their combination for predicting occult PM. These five prediction models achieved an AUC value of 0.832, 0.70, 0.784, 0.838, and 0.941, respectively. The DeLong test and Decision Curve Analysis (DCA) showed that the joint model, containing three meaningful CT signs (maximum size, thickness, and ascites) and two meaningful texture parameters (inhomogeneity of the primary tumor and inhomogeneity of the peritoneum), possessed the best predictive performance and clinical application (*p*<0.05). A forecast nomogram was subsequently established from the model above-mentioned. The calibration curves of the nomogram indicated a good consistency (a concordance index of 0.807) between the projection and the actual observation of occult PM.

**Conclusions:**

A practical projection nomogram based on the comprehensive CT texture analysis of a primary tumor and peritoneotome combined with conventional CT signs was constructed in our study, which can be conveniently used in preoperative personalized prediction of occult PM for GC patients, and acts as a recommendation for the optimization of clinical management.

## Introduction

Gastric cancer (GC) is one of the leading causes of death in China. It is the fifth most common malignancy and the third deadliest tumor in the world, and it remains a major challenge to public health on a global scale (1–3). Moreover, peritoneal metastasis (PM) is the most often pattern of metastasis and recurrence in GC patients, which means a poor prognosis (4, 5).

Occult PM is defined as PM that cannot be preoperatively recognized using conventional computed tomography (CT) until it is diagnosed after surgery or pathological results, which may cause patients to undergo unnecessary surgical injury due to the difficulty of obtaining the most vivid imaging interpretation about occult PM before the elective surgery. This often leads to a precarious situation because of the uncertain state concerning occult PM (6). Thus, preoperative knowledge of occult PM is vital for making treatment plan and prognosis assessment in GC patients.

Nevertheless, CT, MRI, and gastroscopy have poor sensitivity to detecting occult PM of GC. Currently, only laparoscopic exploration is able to diagnose peritoneal micrometastases. However, because it is an invasive and costly procedure, it has not been widely used in clinical practice (7).

A great deal of imaging studies has revolved around the PM state in GC. CT should be the most appropriate image evaluation method for detecting PM (8) because it can noninvasively and comprehensively evaluate the lesion and its adjacent structures for the preoperative stage of GC. Typical signs shown by CT such as omentum cake, parietal peritoneal thickening, and ascites are an easy diagnosis for PM in GC; but these indicate poor prognosis. Although the interpretation of CT signs by the naked eye provides useful information, some hidden image signs related to the clinical findings might be ignored due to the limited image contrast of the naked eye. It is limited in performing a further accurate assessment of occult PM (9, 10). Therefore, identifying a noninvasive and preoperative evaluation approach to detecting occult PM in GC would be vital in order to avoid unnecessary surgery and select the best treatment option in clinical practice.

Texture analysis is a type of comprehensive medical image analysis that is now at a stage of rapid progress. It is applied when performing quantitative analysis of tumor heterogeneity by analyzing the distribution and the relationship of pixels or voxel gray levels in the target region. The ease of obtaining texture information from routinely acquired images without additional imaging procedures is required, and accumulating data showing the correlation between heterogeneity and adverse tumor biology is a major advantage of the technique (11). Preliminary studies report that texture analysis, as a noninvasive imaging tool, has a great potential in predicting the histopathological grade (12), the overall survival (13–16), and the response to neo-adjuvant therapy of gastric cancer (14, 15, 17). Recently, some studies have shown that radiomics analysis based on CT can help diagnose PM in GC (10, 11, 18). However, only texture parameters extracted from the CT images of the largest cross-sectional area of the omentum (10) or the area of greatest enhancement (11) or the nearby peritoneum (18) cannot comprehensively evaluate occult PM.

Therefore, it may be possible to provide more information for occult PM by excavating the CT images of the whole peritoneal and primary tumor of GC patients, which is an interesting issue that deserves further research.

Hence, in order to judge the independent predictor of occult PM more comprehensively, our study aims to retrospectively investigate whether a nomogram model based on texture analysis of the whole peritoneal area combined with the entire primary tumor and conventional CT signs can preoperatively predict occult PM in GC patients.

## Material and methods

This retrospective study was approved by the institutional review board of Fujian Province Hospital, and the requirement of patients’ informed consent was waived.

### Patients

**Figure 1:**
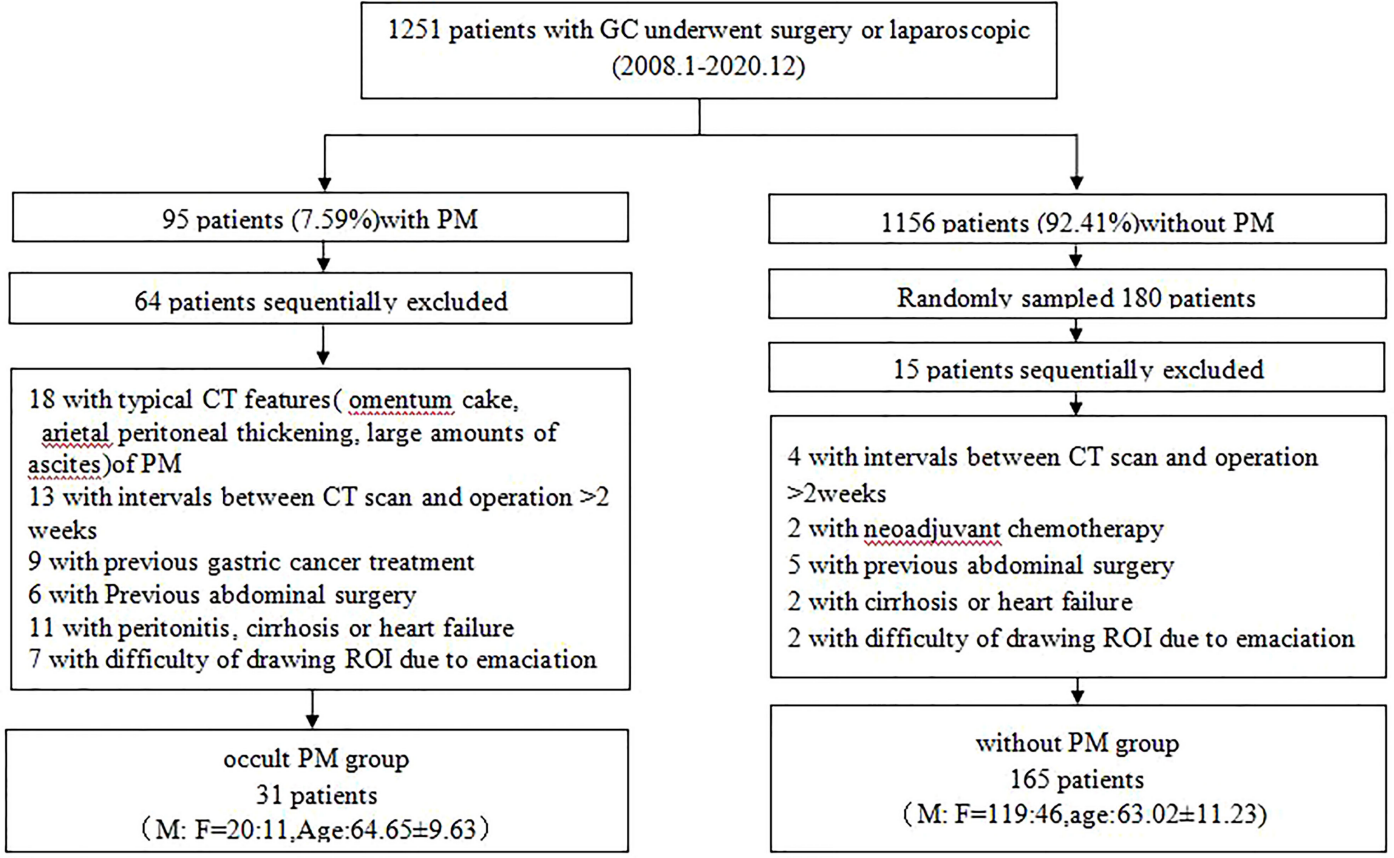
Flowchart of study subject selection.

A total of 1,251 patients with GC who underwent surgery or laparoscopic exploration at our hospital from January 2008 to December 2020 were analyzed retrospectively. Among them, 95 (7.59%) were found to have PM on histologic examination. A total of 196 patients with GC were enrolled (139 men and 57 women, mean age of 70.86 years old, range from 25 to 90 years old), in which 31 cases were occult PM. The flowchart (**Figure 1**) shows the summary of the enrollment of the patients.

### CT image protocol

Patients generally underwent contrast-enhanced CT of the entire abdomen, which is from the dome of the diaphragm to the pubic symphysis according to the standard clinical scanning protocols (tube voltage of 120 kV, tube current of 180–200 mAs, slice thickness of 3 mm, slice interval of 3 mm, field view of 35–40 cm, matrix of 256 × 256, and pitch of 0.6–1.0) on CT machines (Somatom Definition AS 128; Somatom Sensation 64; Light speed 128, GE Healthcare). All CT scans were reconstructed into slices of 3-mm thickness and interval. After fasting from food for at least 6 h, each patient ingested about 600–1000 ml of water for more than 15 min to achieve gastric distension before scanning. All patients were trained to hold their breath in the supine position during CT scanning. After the plain CT, a 1.5 ml/kg iodinated contrast agent (ioversol 320 mgI/mL, Jiangsu hengrui) was injected with a pump injector (OptiVantage DH, Tyco) at a flow rate of 3 ml/s into the dorsal hand vein. The CT images were obtained during the arterial phase (25–30 s after the initiation of the injection), the portal venous phase (60–70 s), and the delay phase (180 s). The acquired images were analyzed on a PACS station (GE Healthcare).

### Image analysis

The portal-venous-phase images were evaluated by two radiologists (Y-xT and H-jZ, with 10 and 20 years of experience in abdominal CT imaging, respectively), who were blinded to the clinical and histopathological data. They retrospectively analyzed and reached a consensus for the following CT signs: GC lesion location (junction, bottom, body, antrum, or diffuse); tumor maximum size (the longest diameter measured at the largest cross-section of the mass); tumor thickness; degree of lesion enhancement (the CT value difference between the venous phase and the plain phase); serosal involvement (rough); enlarged lymph nodes (the short-axis diameter is more than 10 mm, regardless of the location); and ascites and definitive CT findings of PM (including omental nodules or cake, irregular thickening of peritoneal with high enhancement). When two physicians had different opinions, a third physician (X-fC) would evaluate the images and take the average value as the result. Discrepancies were resolved through a consensus after the joint reevaluation of the images.

### CT image texture features extraction

**Figure 2:**
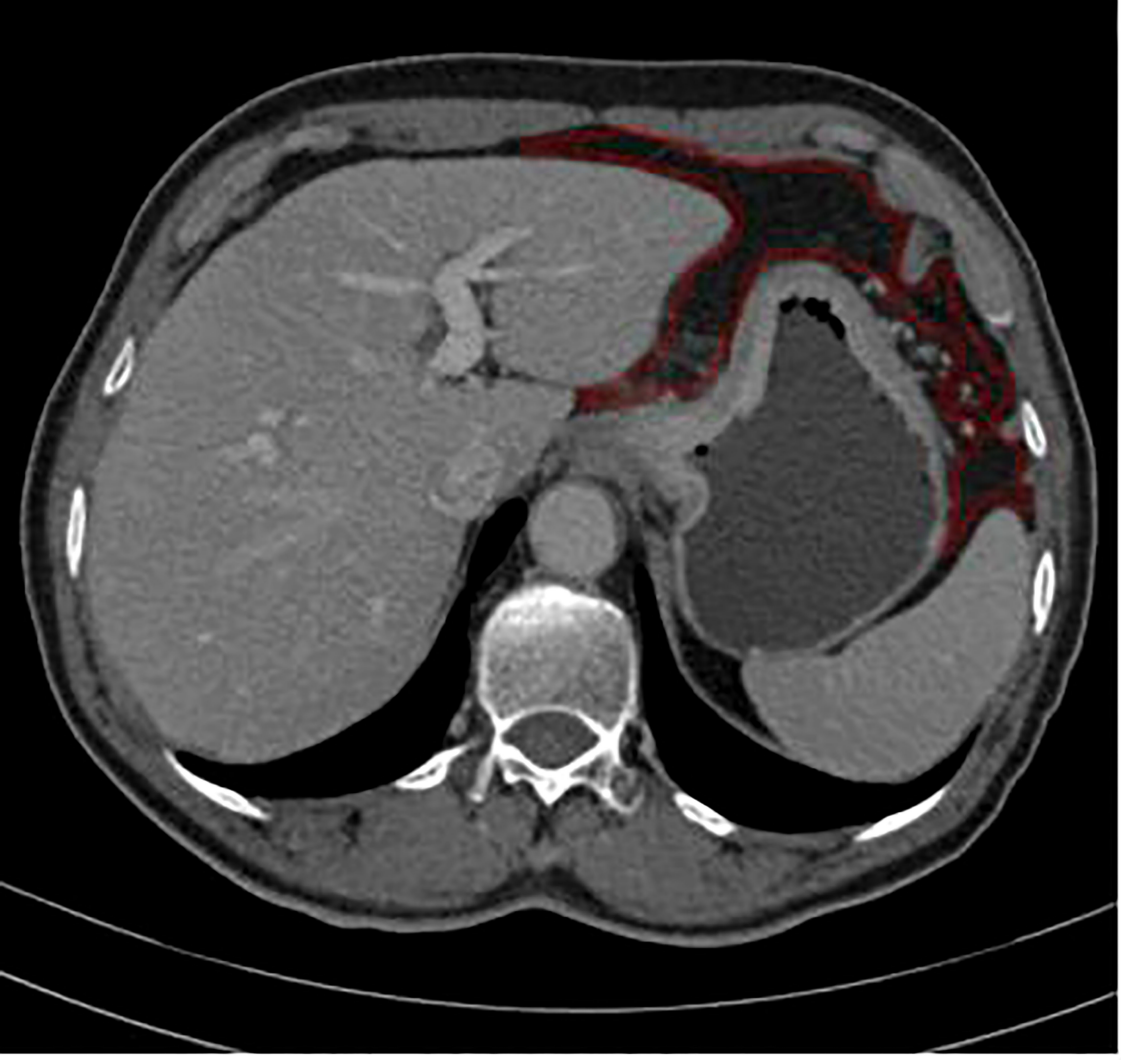
ROI annotation (red line) on a representative abdomen enhanced venous CT image, as an example of drawning ROI covering the peritoneotome or primary lesion slice by slice.

Portal-venous-phase CT images were exported to firevoexl software (https://firevoxel.org) for texture analysis. The lesions of GC and peritoneotome were manually recognized under the consensus of two radiologists who were both blinded to the clinical and pathological information of the patients. They reviewed all slices of the CT images and manually drew the polygonal region of interest (ROI) along the margin of the GC lesion and the peritoneotome slice by slice; the gastric lumen, blood vessels, other organs, and artifacts were carefully avoided (**Figure 2**). The texture feature parameters, including the mean, standard deviation, inhomogeneity, skewness, kurtosis, and entropy, were extracted automatically from the delineated ROIs.

### PM status ascertainment

All patients enrolled underwent surgery or laparoscopic exploration. Any suspect lesion discovered during surgery or laparoscopy was biopsied and examined pathologically to confirm the PM status. Occult PM was defined as PM that could not be preoperatively detected on CT until it was confirmed pathologically.

### Statistical analysis

Data analysis was performed using SPSS 22.0 (IBM, Armonk, NY, USA), MedCalc 15.2.2, and R software version 3.6.3 (R Foundation for Statistical Computing, Vienna, Austria, http://www.Rproject.org). A two-tailed *p* value of <0.05 indicated statistical significance. The Mann–Whitney U test was used to assess the differences in continuous variables, whereas the Wilcoxon rank-sum and chi-square tests or Fisher’s exact test was used for categorical variables.

### Factor selection and prediction model construction

Univariate analysis was applied to clinical features, CT signs, and texture characteristics to discern the dependent predictors of occult PM.

The receiver operating characteristic (ROC) curves were applied to assess all continuous variables and then were divided into different subgroups according to the cutoff values. Subsequently, statistically significant predictors were included in the stepwise logistic multivariate regression and were used as the parameters to build the prediction model.

### Development and validation of prediction models

The efficiency of the models was tested by the AUC of the ROC curve and compared by the DeLong test in MedCalc.

Independent risk factors for occult PM were confirmed by multivariate logistic regression analysis as mentioned above and were used to create a model that graphically represents these risk factors. To provide a visually quantitative tool to predict occult PM in GC patients, subsequently, we developed a nomogram based on the prediction model with the best AUC value and clinical utility. Moreover, we constructed a calibration plot to reduce the overfit bias of the nomogram.

## Results

### Patient characteristics

A total of 196 consecutive patients with gastric cancer who underwent surgery or laparoscopy in our institution were included in the retrospective study between January 2008 and December 2020, as shown in the flowchart of the study subject selection (**Figure 1**). Among them, 31 were found to have occult PM on histologic examination. Patients and tumor characteristics are summarized in **Table 1**.

**Table 1:** Univariate analysis of the clinical, pathological, and texture characteristics of gastric cancer patients.

Factors			Without PM	Occult PM	*P*
	Number of patients		165 (84.2 %)	31 (15.8 %)	
Clinical	Sex	Male	119 (72.1 %)	20 (64.5%)	0.392
		Female	46 (27.9 %)	11 (35.5 %)	
	Age (Mean ± SD) years		63.02±11.23	64.65±9.625	0.583
Pathological	Differentiation degree	Well	79 (47.9 %)	9 (29 %)	0.031
		Moderately	17 (10.3 %)	1 (3.2 %)	
		Poorly	69 (41.8 %)	21 (67.7 %)	
	Lauren type	Intestinal type	149 (90.3 %)	25 (80.6 %)	0.127
		Mixed and Diffuse type	16 (9.7 %)	6 (19.4 %)	
	Serous	–	112 (67.9 %)	2 (6.5 %)	<0.001
		+	53 (32.1 %)	29 (93.5 %)	
	Lymph node metastasis	–	76 (46.1 %)	12 (38.7 %)	0.450
		+	89 (53.9 %)	19 (61.3 %)	
CT signs	Lesion location	Junction	39 (23.6 %)	4 (12.9%)	0.622
		Bottom	8 (4.8 %)	2 (6.5 %)	
		Body	49 (29.7 %)	12 (38.7 %)	
		Antrum	60 (36.4 %)	11 (35.5 %)	
		Diffuse	9 (5.5 %)	2 (6.5 %)	
	Primary tumor max size	<=3.55cm	85 (51.5 %)	5 (16.1 %)	<0.001
		>3.55cm	80 (48.5 %)	26 (83.9 %)	
	Primary tumor thickness	<=1.85cm	118 (71.5 %)	14 (45.2 %)	0.004
		>1.85cm	47 (28.5 %)	17 (54.8 %)	
	Primary tumor enhancement	<=53.5Hu	117 (70.9 %)	14 (45.2 %)	0.007
		>53.5Hu	48 (29.1 %)	17 (54.8 %)	
	CT serous	–	59 (35.8 %)	6 (19.4 %)	0.075
		+	106 (64.2 %)	25 (80.6 %)	
	CT lymph node	–	162 (98.2%)	28 (90.3 %)	0.053
		+	3 (1.8%)	3 (9.7%)	
	Ascites	–	166 (98.2%)	25 (80.6%)	0.001
		+	3 (1.8%)	6 (19.4%)	
Primary tumor texture	StDev	<=34.72	98 (59.4%)	8 (25.8%)	0.001
		>34.72	67 (40.6 %)	23 ( 74.2%)	
	Inhomogeneity	<=0.034	95 (57.6%)	7 (22.6%)	<0.001
		>0.034	70 (42.4%)	24 (77.4%)	
	Entropy	<=3.23	107 (64.8%)	27 (87.1%)	0.015
		>3.23	58 (35.2%)	4 (12.9%)	
Peritoneum texture	StDev	<=28.69	130 (78.8 %)	9 (29%)	<0.001
		>28.69	35 (21.2%)	22 ( 71%)	
	Inhomogeneity	<=0.034	156 (94.5%)	15 (48.4 %)	<0.001
		>0.034	9 (5.5 %)	16 (51.6 %)	

-: negative, +: positive.

Clinical, pathological, and texture characteristics of patients are shown in **Tables 1**–**3**. There is a significant difference (*p*< = 0.05) between the occult PM group and the group without PM in terms of the differentiation degree and serosal involvement as confirmed by pathology; CT signs of a primary tumor in terms of size, thickness, enhancement, and ascites; primary tumor texture features of standard deviation, inhomogeneity, and entropy; and peritoneum texture features of standard deviation and inhomogeneity. On the other hand, no significant difference (*p*> = 0.05) was found between the two groups with regard to sex; age; pathological and Lauren feature types; lymph node metastasis as confirmed by pathology; primary tumor texture features of the mean, skewness, and kurtosis; and peritoneum texture features of the mean, skewness, kurtosis, and entropy.

**Table 2:** Receiver operating characteristic curves of every continuous variable.

Variable	Cut off	*P*	AUC	Sensitivity	Specificity	95%CI
Primary tumor max size	3.55	<0.001	0.722	83.9	51.5	0.628–0.816
Primary tumor thickness	1.85	0.005	0.66	54.8	71.5	0.559–0.76
Primary tumor enhancement	53.5	0.053	0.662	54.8	70.9	0.558–0.766
Primary tumor StDev	34.72	0.005	0.661	77.4	59.4	0.59–0.727
Primary tumor Inhomogeneity	0.034	0.003	0.668	80.6	54.5	0.597–0.734
Primary tumor Entropy	3.23	0.026	0.626	35.2	87.1	0.555–0.694
Peritoneum StDev	28.69	<0.001	0.791	71	78.8	0.728–0.846
Peritoneum inhomogeneity	0.034	<0.001	0.78	51.6	94.5	0.716–0.836

**Table 3:** Variables and coefficients of CT signs and texture features model.

Variable	CT signs model			Primary tumor texture model			Peritoneum texture model		
	B	Adjusted OR (95% CI)	*P*	B	Adjusted OR (95% CI)	*P*	B	Adjusted OR (95% CI)	*P*
Intercept	-5.072	0.006	<0.001	-4.145	0.043	0.000	-5.916	0.003	<0.001
Tumor max size (>3.55 vs.<=3.55)	2.024	7.568 (2.373–24.131)	0.001	\	\	\	\	\	\
Thickness (>1.85 vs.<=1.85)	1.018	2.769 (1.134–6.756)	0.025	\	\	\	\	\	\
Enhancement (>53.5 vs.<=53.5)	0.897	2.453 (1.009–5.967)	0.048	\	\	\	\	\	\
Ascites (andvs-)	2.977	19.632 (3.713–103.805)	<0.001	\	\	\	\	\	\
CT serous (andvs-)	1.292	3.639 (1.119–11.838)	0.032	\	\	\	\	\	\
Primary tumor Inhomogeneity (>0.034 vs. <=0.034)	\	\	\	1.538	4.653 (1.898~11.408)	0.001	\	\	\
Peritoneum StDev (>28.69 vs. <=28.69 )	\	\	\	\	\	\	1.204	3.333 (1.0926–10.17)	0.034
Peritoneum Inhomogeneity (>0.034 vs. <=0.034 )	\	\	\	\	\	\	2.042	7.704 (2.306–25.736)	0.001
**Variable**	**Total Texture model**			**Total Texture and CT signs model**					
	**B**	**Adjusted OR (95% CI)**	***P***	**B**	**Adjusted OR (95% CI)**	***P***			
Intercept	-3.301	0.037	<0.001	-6.44	0.002	<0.001			
Tumor max size (>3.55 vs. <=3.55)	\	\	\	2.558	12.905 (2.976–55.969)	0.001			
Thickness (>1.85 vs. <=1.85)	\	\	\	1.454	4.282 (1.361–13.47)	0.013			
Enhancement (>53.5 vs. <=53.5)	\	\	\						
Ascites (andvs-)	\	\	\	4.238	69.282 (7.19–667.558)	<0.001			
CT serous (andvs-)	\	\	\	\	\	\			
Primary tumor inhomogeneity (>0.034 vs. <=0.034)	1.565	4.785 (1.715–13.354)	0.000	1.997	7.367 (2.057–26.384)	0.002			
Peritoneum StDev (>28.69 vs. <=28.69 )	\	\	\	\	\	\			
Peritoneum inhomogeneity (>0.034 vs. <=0.034 )	2.939	18.889 (6.629–53.821)	0.000	3.844	46.693 (10.433–208.974)	<0.001			

### ROC curves of continuous variables

**Figure 3:**
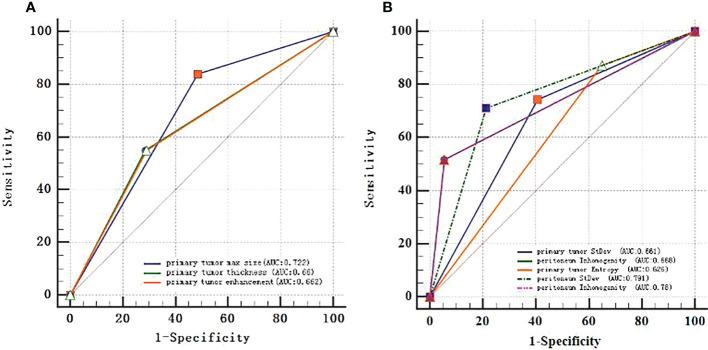
ROC curves of every continuous variable. **(A)**, primary tumor CT signs. **(B)**, texture features. The area under the curve (AUC) represented the accuracy of predicting for occult PM.

The ROC curves were applied to evaluate the continuous variables. The cutoff value for the ROC curve was determined by the Youden index, in which the highest Youden index was the most optimal level to discriminate the occult PM group from the group without PM, as shown in **Table 2**. Regarding the ROC curves for the maximum size, thickness, enhancement, standard deviation, inhomogeneity, and entropy of the primary tumor, the cutoff values were 3.55 cm, 1.85 cm, 53.5 Hu, 34.72, 0.034, and 3.23, respectively [with areas under the curve (AUC) values of 0.722, 0.66, 0.662, 0.661, 0.668, and 0.626, respectively]; whereas for the standard deviation and inhomogeneity of the peritoneum, the cutoff values were 28.69 and 0.034, respectively (with AUC values of 0.791 and 0.78, respectively). Exhaustive results such as AUC (95%CI), sensitivity, and specificity are shown in **Table 2** and **Figure 3**. The primary tumor texture features standard deviation, inhomogeneity, and entropy and the peritoneum texture features standard deviation and inhomogeneity were significant predictors according to the ROC curve. There was no statistically significant difference in the other indicators.

### Establish different models

A total of five CT signs and three texture features were extracted from enhanced CT images during the venous phase of the 196 GC patients, and there was a good consistent agreement between the two radiologists (readers 1 and 2) for the texture features (all ICCs > 0.8, p < 0.05). Then, the average measurement value from the two radiologists was applied for further investigation.

The preoperative CT characteristic features included the CT signs of the primary tumor size, thickness, enhancement, and ascites; the primary tumor texture features standard deviation, inhomogeneity, and entropy; and the peritoneum texture features standard deviation and inhomogeneity, which are shown in **Table 1**; they were used in the multivariable logistic regression analysis to confirm the independent predictors of occult PM. The results of the multivariate logistic analysis showed that the CT signs of serosal involvement, ascites, and size, thickness, and enhancement of the primary tumor; the primary tumor texture features standard deviation, inhomogeneity, and entropy; and the peritoneum texture features standard deviation and inhomogeneity were independent predictive factors for occult PM (p < 0.05). The outcomes are shown in **Table 3**. Thus, we integrated these factors into the CT signs model, the primary tumor texture model, the peritoneum texture model, the primary tumor texture + peritoneum texture (Total Texture) model, and the Total Texture + CT signs model to predict occult PM in GC patients.

The diagnostic performance of the model was assessed using the ROC curve. The Total Texture model showed slightly better diagnostic efficacy than the primary tumor texture model and the peritoneum texture model (AUC, 0.838 vs.0.7 and 0.784; sensitivity, 0.677 vs. 0.774 and 0.71; specificity, 0.873 vs. 0.576 and 0.788, respectively) as shown in **Table 4**. However, the comparison of ROC curves showed that Total Texture vs. peritoneum texture, Total Texture vs. CT signs, Total Texture + CT signs vs. CT signs, peritoneum texture vs. primary tumor texture, and peritoneum texture vs. CT signs were not significant (*p*> = 0.05). There was no statistical significance between peritoneum texture, primary tumor texture, and Total Texture + CT signs in diagnosing occult PM (*p*> = 0.05). On the other hand, the Total Texture + CT signs model based on the whole peritoneal area texture analysis combined with the entire tumor and conventional CT signs showed better diagnostic efficacy than the CT signs, primary tumor texture, peritoneum texture, and Total Texture models (*p*<0.05). Which are shown in **Table 5**.

**Table 4:** ROC curves parameters of different models.

Variable	Cutoff Value	*P*	AUC	Sensitivity	Specificity	95% CI
CT signs	0.244	0.000	0.832	0.774	0.818	0.772–0.882
Primary tumor texture	0.124	0.000	0.70	0.774	0.576	0.631–0.763
Peritoneum texture	0.126	0.000	0.784	0.71	0.788	0.720–0.840
Total Texture	0.275	0.000	0.838	0.677	0.873	0.779–0.886
Total Texture + CT signs	0.116	0.000	0.941	0.935	0.794	0.899–0.970

Total Texture refers to primary tumor texture + peritoneum texture.

**Table 5:** Comparison of ROC curves AUC areas of different models.

Different models	Difference of AUC areas	*Z*	*P*
Total Texture vs. primary tumor texture	0.138	3.261	0.0011
Total Texture vs. peritoneum texture	0.054	1.896	0.058
Total Texture vs. CT signs	0.0055	0.084	0.9331
Total Texture vs. Total Texture CT signs	0.104	3.117	0.018
Total Texture CT signs vs. primary tumor texture	0.241	6.094	0.0001
Total Texture CT signs vs. peritoneum texture	0.157	3.946	0.0001
Total Texture CT signs vs. CT signs	0.109	2.76	0.0058
Peritoneum texture vs. peritoneum texture	0.084	1.385	0.1661
Peritoneum texture vs. CT signs	0.0481	0.712	0.476
Primary tumor texture vs. CT signs	0.132	2.15	0.0316

Total Texture refers to primary tumor texture + peritoneum texture.

The Total Texture + CT signs, Total Texture, and CT signs models have considerable predictive capabilities, with a C-index of 0.807, 0.917, and 0.829, an AUC value of 0.941, 0.838, and 0.832, and a 95% confidence interval of 0.899−0.970, 0.779−0.886, and 0.772−0.882, respectively. Which are shown in **Table 6**. 

**Table 6:** Parameters of calibration curves of different prediction models.

Model	C-Indexes	Brier
fit1 represents CT signs model for green	0.829	0.099
fit2 represents primary tumor texture model for blue	0.675	0.124
fit3 represents peritoneum texture model for red	0.784	0.097
fit4 represents Total Texture model for yellow	0.917	0.067
fit5 represents Total Texture and CT signs model for pink	0.807	0.091

**Figure 4:**
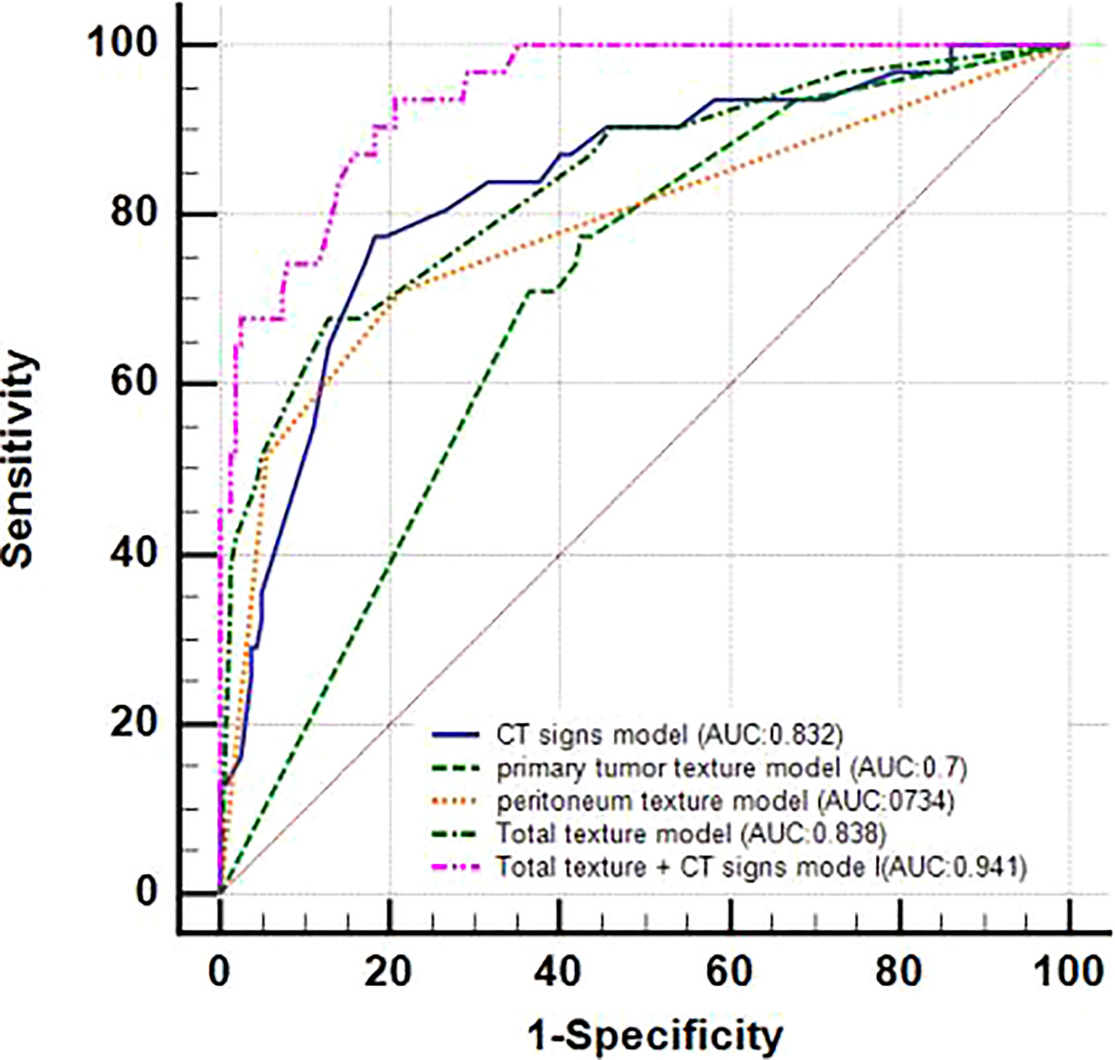
ROC curves of 5 different prediction models.

**Figure 5:**
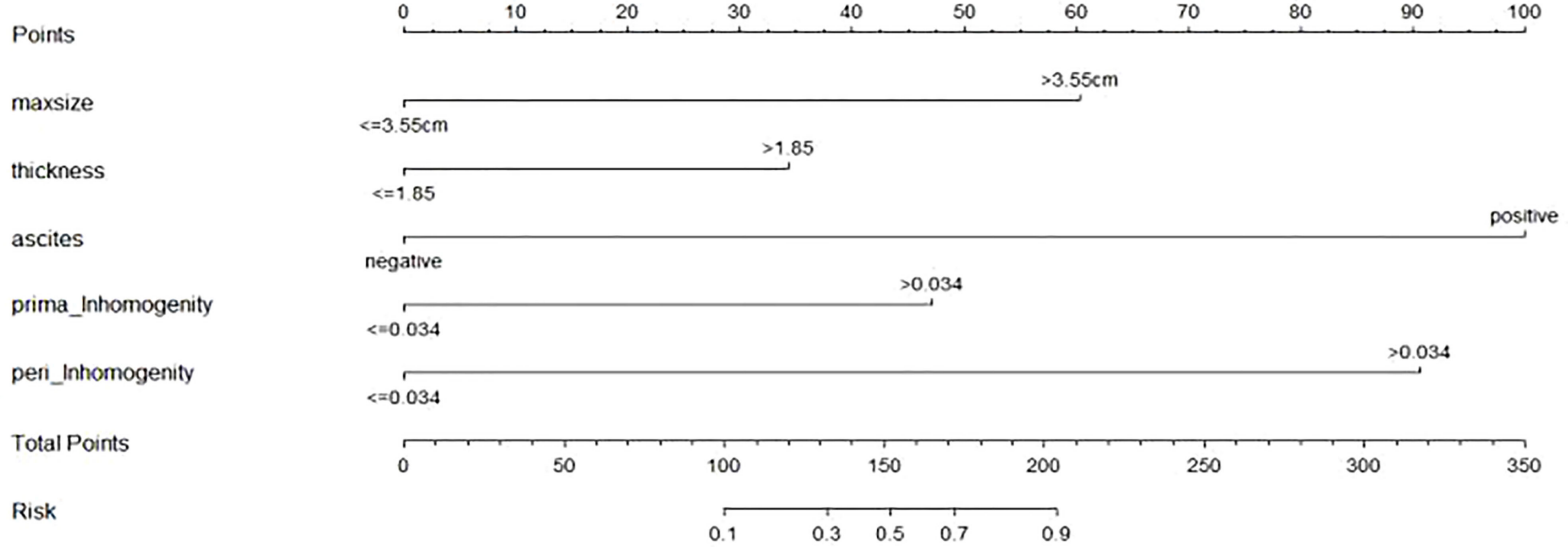
Developed prediction nomogram based on primary tumor Texture + peritoneum Texture + CT signs model. The probability of each predictor can be converted into scores according to the first scale "Points" at the top of the nomogram. Total points are calculated by adding up the point value for each predictor, which is determined by drawing a line straight upward to the total points axis at the bottom of the nomogram, than draw a line straight down to the risk axis to determine the possibility of occult PM in patients with gastric cancer.

**Figure 6:**
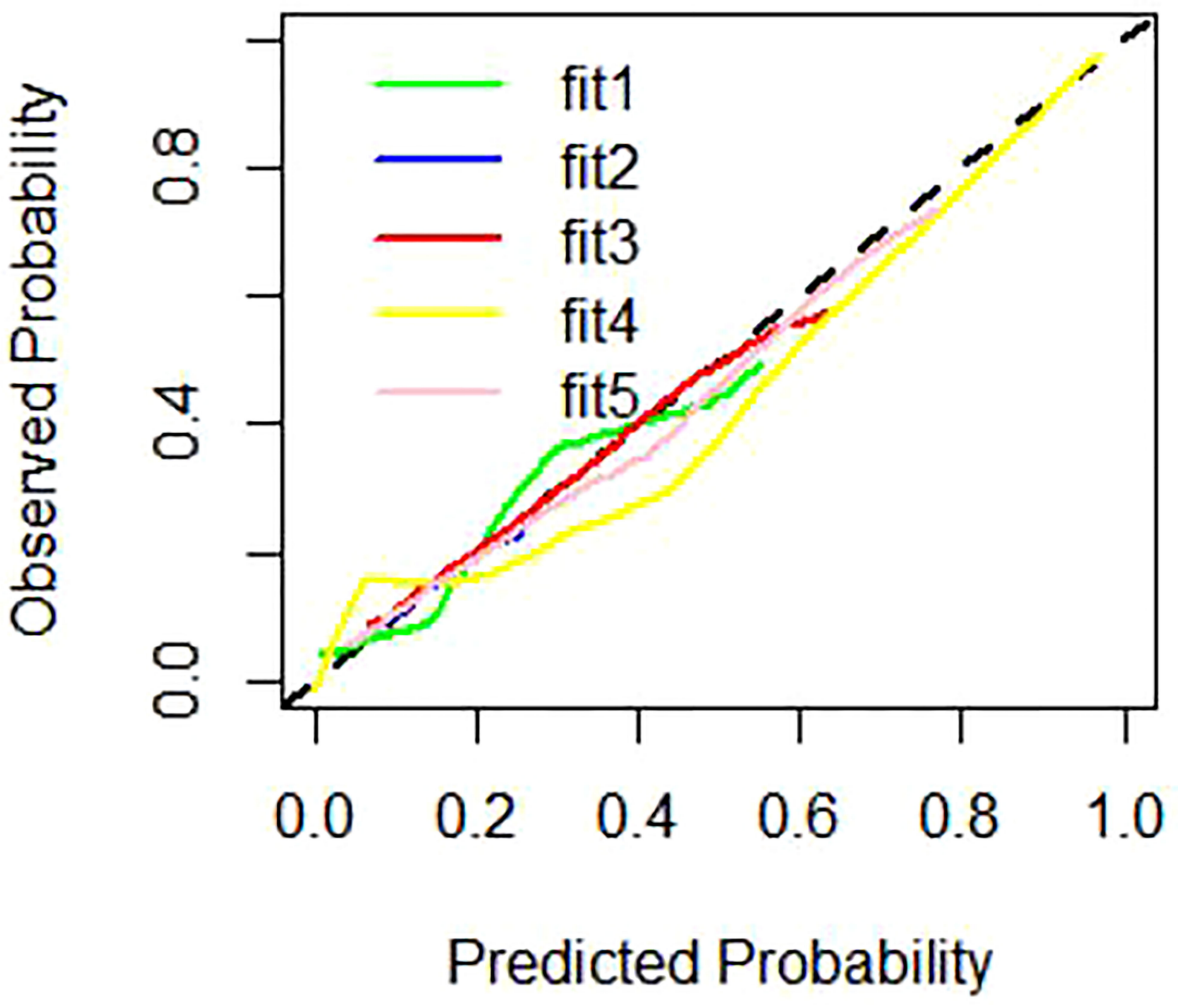
Calibration curves of different prediction models. Note: fitl represent CT signs model for green line, fit2 represent primary tumor texture model for blue line, fit3 represent peritoneum texture model for red line, fit4 represent Total Texture model for yellow line, fit5 represent Total Texture+ CT signs model for pink line.

The nomogram includes risk factors that may predict the likelihood of PM, as shown in **Figures 3**–**5**. The total risk score for occult PM was the sum of the scores for these risk factors. The corresponding value on the risk axis is the probability that the patient may develop PM. Moreover, we developed an internal calibration curve to evaluate the predictive accuracy of the nomogram and found that the C-index was 0.829, 0.675, 0.784, 0.917, and 0.807 for the CT signs, primary tumor texture, peritoneum texture, Total Texture, and Total Texture + CT signs models, respectively, indicating a good fit (**Figure 6**).

## Discussion

In the present study, we separately developed five prediction models using CT signs, primary tumor texture, peritoneum texture, primary tumor texture + peritoneum texture, and their combination to predict occult PM. The DeLong test and DCA indicate that the combined model, consisting of three meaningful CT signs (maximum size, thickness, and ascites) and two meaningful texture parameters (inhomogeneity of the primary tumor and inhomogeneity of the peritoneum), held the best predictive efficiency and clinical utility (p < 0.05). A quantitative prediction nomogram was accordingly established based on the combined model. The calibration curves revealed a nice consistency between the actual results and the nomogram predictions of occult PM in GC patients.

The peritoneum is the most frequent site where GC spreads, and this is strongly associated with poor prognosis. The classic theory of tumor metastasis is the “seed-and-soil” theory (19), which takes into account the fact that PM in GC initiation depends on the synergistic effect of the primary tumor and the peritoneal microenvironment and progresses through the following steps: the tumor cells depart from the primary tumor, attach to the distant peritoneum, and invade the subperitoneal space, which eventually develop to hyperplasia and then to angiogenesis (7, 19). The results of our study are consistent with this assumption and indicate that occult PM can be evaluated more comprehensively by combining the texture features of the entire tumor, the entire area of the peritoneum, and conventional CT findings, which can more comprehensively determine the independent predictors of PM.

Recently, many CT radiomics studies focused on the PM of GC. Liu et al. (12) reported that venous CT radiomics analysis based on the primary tumor provided valuable information for predicting occult PM in advanced GC (AGC). Meanwhile, Kim et al. (11) found that CT texture features over the omentum, that is, entropy, held potential promise in distinguishing patients with occult PM and those without occult PM. In another study, Dong et al. (18) found that venous CT radiomics analysis combining both the primary tumor and the nearby peritoneum had an excellent prediction value of occult PM in AGC. On the other hand, in our study, we did not delineate the peritoneal ROIs as Kim et al. (11), Dong et al. (18), or Liu et al. (12) did; we drew the ROI covering the peritoneotome and primary lesion slice by slice, which made it possible to evaluate occult PM more comprehensively, and our study indicated that CT texture analysis over the whole peritoneum, especially the features standard deviation and inhomogeneity, is perhaps a useful aid for the prediction of occult PM in AGC. Moreover, outlining ROIs over the omentum in only one cross-sectional slice is less comprehensive and repetitive. For the following reasons, the PM detected by surgeons not only was limited to a nodule discovered in the omentum but also included anywhere including the peritoneum, cul-de-sac, or mesentery rather than at the maximum cross-section of the omentum (11) or the area of greatest enhancement or the nearby peritoneum (18) that was selected for texture analysis. The analysis of the entire lesion might not only be more representative of the heterogeneous features of the lesion but also improve the repeatability and reproducibility.

The advantages of the nomogram based on the combined model include the following: it provides convenience in clinical application to preoperatively identify occult PM; it serves as a reference for optimizing clinical management, such as regular follow-up; and the progression may be suggested. In addition, patients with a high risk of occult PM who were filtered by the nomogram may benefit from the preoperative neoadjuvant therapy. This can reduce the cost of subsequent diagnosis and help avoid improper surgical procedures, develop more reasonable and effective treatment plans, such as determining optimal candidates for laparoscopy exploration, and prevent patients from having a poor prognosis, which is significant to improving patients’ prognosis and quality of life.

However, although the results are encouraging, there are several limitations of the study. First, CT images were retrospectively analyzed and obtained from multiple scanners, and the inconsistency in scanning the parameters might affect the feature extraction and cause some measurement bias for the analysis. Notwithstanding, a good interscanner agreement of the CT texture analysis was confirmed when using different scanners with different vendors and acquisition processes (20). Second, we delineated the peritoneal ROIs at all levels. This reduces the sampling error and can include not only PM in occult sites such as the peritoneal recess but also areas without PM, obtaining texture parameters that represent the average of the entire peritoneal region. However, patients with excessive weight loss were excluded due to limited ROI delineation, which may have selection bias (20). In addition, we performed texture analysis on venous phase images since the venous stage CT images of the tumor tissue had better contrast with adjacent normal organizational structure (21). Since we conjecture that different delay times of enhancement may influence texture features and their diagnostic performance, in our study, early arterial phase images were obtained with a delay time from 25 to 30 s, which requires further investigation. Third, since retrospective data were used to build the models, some clinical factors, such as the serum tumor marker, were not initially available when incomplete data were considered. This also makes it difficult to apply in emaciated patients lacking a recognizable peritoneum. Eventually, external validation is needed to assess the diagnostic performance and the practicality of the model in different medical institutions.

Nevertheless, despite these limitations, CT texture analysis over the peritoneum and the primary tumor still deserves further investigation. We believe that this nomogram can still help clinicians to develop personalized treatment options for patients with GC and may have significant clinical implications in the early preoperative diagnosis of GC PM and the development of a personalized treatment plan.

## Data availability statement

The original contributions presented in the study are included in the article/supplementary material. Further inquiries can be directed to the corresponding author.

## Ethics statement

The studies involving human participants were reviewed and approved by the institutional review board of Fujian Province Hospital. Written informed consent for participation was not required for this study in accordance with the national legislation and the institutional requirements. Written informed consent was not obtained from the individual(s) for the publication of any potentially identifiable images or data included in this article.

## Author contributions

S-xC, H-jZ, and HW conceived of and designed the study. S-xC drafted the original paper. S-xC, H-jZ, HW, Y-xT, and X-fC extracted all data and performed the statistical analysis based on the data. S-xC and H-jZ supervised the project and provided direction and guidance throughout the preparation of this manuscript. S-xC revised the final manuscript. All authors contributed to the article and approved the submitted version.

## Funding

This research was funded by Startup Fund for scientific research, Fujian Medical University [grant number: 2018QH1138]and Guiding project of Science and Technology of Fujian Province [grant number 2022Y0050].

## Conflict of interest

The authors declare that the research was conducted in the absence of any commercial or financial relationships that could be construed as a potential conflict of interest.

## Publisher’s note

All claims expressed in this article are solely those of the authors and do not necessarily represent those of their affiliated organizations, or those of the publisher, the editors and the reviewers. Any product that may be evaluated in this article, or claim that may be made by its manufacturer, is not guaranteed or endorsed by the publisher.

## References

[1] Arnold M Park JY Camargo MC Lunet N Forman D Soerjomataram I. Is gastric cancer becoming a rare disease? a global assessment of predicted incidence trends to 2035. Gut (2020) 69:823–9. doi: 10.1136/gutjnl-2019-320234 PMC8520492 32001553

[2] Parisi A Porzio G Ficorella C. Multimodality treatment in metastatic gastric cancer: From past to next future. Cancers (Basel) (2020) 12:2598. doi: 10.3390/cancers12092598 32932914 PMC7563615

[3] Sung H Ferlay J Siegel RL Laversanne M Soerjomataram I Jemal A. Global cancer statistics 2020: GLOBOCAN estimates of incidence and mortality worldwide for 36 cancers in 185 countries. CA Cancer J Clin (2021) 71:209–49. doi: 10.3322/caac.21660 33538338

[4] Seyfried F von Rahden BH Miras AD Gasser M Maeder U Kunzmann V. Incidence, time course and independent risk factors for metachronous peritoneal carcinomatosis of gastric origin–a longitudinal experience from a prospectively collected database of 1108 patients. BMC Cancer (2015) 15:73. doi: 10.1186/s12885-015-1081-8 25879885 PMC4337241

[5] Spolverato G Ejaz A Kim Y Squires MH Poultsides GA Fields RC. Rates and patterns of recurrence after curative intent resection for gastric cancer: a united states multi-institutional analysis. J Am Coll Surg (2014) 219:664–75. doi: 10.1016/j.jamcollsurg.2014.03.062 25154671

[6] Kitayama J Ishigami H Yamaguchi H Sakuma Y Horie H Hosoya Y. Treatment of patients with peritoneal metastases from gastric cancer. Ann Gastroenterol Surg (2018) 2:116–23. doi: 10.1002/ags3.12060 PMC5881364 29863151

[7] Sun F Feng M Guan W. Mechanisms of peritoneal dissemination in gastric cancer. Oncol Lett (2017) 14:6991–8. doi: 10.3892/ol.2017.7149 PMC5754894 29344127

[8] Huang Z Liu D Chen X Yu P Wu J Song B. Retrospective imaging studies of gastric cancer: Study protocol clinical trial (SPIRIT compliant). Med (Baltimore) (2020) 99:e19157. doi: 10.1097/MD.0000000000019157 PMC7034669 32080093

[9] Lee IS Lee H Hur H Kanda M Yook JH Kim BS. Transcriptomic profiling identifies a risk stratification signature for predicting peritoneal recurrence and micrometastasis in gastric cancer. Clin Cancer Res (2021) 27:2292–2300. doi: 10.1158/1078-0432.CCR-20-3835 PMC8103893 33558424

[10] Liu S He J Liu S Ji C Guan W Chen L. Radiomics analysis using contrast-enhanced CT for preoperative prediction of occult peritoneal metastasis in advanced gastric cancer. Eur Radiol (2020) 30:239–46. doi: 10.1007/s00330-019-06368-5 31385045

[11] Kim HY Kim YH Yun G Chang W Lee YJ Kim B. Could texture features from preoperative CT image be used for predicting occult peritoneal carcinomatosis in patients with advanced gastric cancer. PloS One (2018) 13:e0194755. doi: 10.1371/journal.pone.0194755 29596522 PMC5875782

[12] Liu S Liu S Ji C Zheng H Pan X Zhang Y. Application of CT texture analysis in predicting histopathological characteristics of gastric cancers. Eur Radiol (2017) 27:4951–9. doi: 10.1007/s00330-017-4881-1 28643092

[13] Giganti F Antunes S Salerno A Ambrosi A Marra P Nicoletti R. Gastric cancer: texture analysis from multidetector computed tomography as a potential preoperative prognostic biomarker. Eur Radiol (2017) 27:1831–9. doi: 10.1007/s00330-016-4540-y 27553932

[14] Jiang Y Jin C Yu H Wu J Chen C Yuan Q. Development and validation of a deep learning CT signature to predict survival and chemotherapy benefit in gastric cancer: A multicenter, retrospective study. Ann Surg (2020) 274:e1153–61. doi: 10.1097/SLA.0000000000003778 31913871

[15] Liu X Wu Z Lin E Li W Chen Y Sun X. Systemic prognostic score and nomogram based on inflammatory, nutritional and tumor markers predict cancer-specific survival in stage II-III gastric cancer patients with adjuvant chemotherapy. Clin Nutr (2019) 38:1853–60. doi: 10.1016/j.clnu.2018.07.015 30075998

[16] Zhang W Fang M Dong D Wang X Ke X Zhang L. Development and validation of a CT-based radiomic nomogram for preoperative prediction of early recurrence in advanced gastric cancer. Radiother Oncol (2020) 145:13–20. doi: 10.1016/j.radonc.2019.11.023 31869677

[17] Giganti F Marra P Ambrosi A Salerno A Antunes S Chiari D. Pre-treatment MDCT-based texture analysis for therapy response prediction in gastric cancer: Comparison with tumour regression grade at final histology. Eur J Radiol (2017) 90:129–37. doi: 10.1016/j.ejrad.2017.02.043 28583623

[18] Dong D Tang L Li ZY Fang MJ Gao JB Shan XH. Development and validation of an individualized nomogram to identify occult peritoneal metastasis in patients with advanced gastric cancer. Ann Oncol (2019) 30:431–8. doi: 10.1093/annonc/mdz001 PMC6442651 30689702

[19] Fidler IJ. The pathogenesis of cancer metastasis: the 'seed and soil' hypothesis revisited. Nat Rev Cancer (2003) 3:453–8. doi: 10.1038/nrc1098 12778135

[20] Ng F Kozarski R Ganeshan B Goh V. Assessment of tumor heterogeneity by CT texture analysis: can the largest cross-sectional area be used as an alternative to whole tumor analysis. Eur J Radiol (2013) 82:342–8. doi: 10.1016/j.ejrad.2012.10.023 23194641

[21] Huang YQ Liang CH He L Tian J Liang CS Chen X. Development and validation of a radiomics nomogram for preoperative prediction of lymph node metastasis in colorectal cancer. J Clin Oncol (2016) 34:2157–64. doi: 10.1200/JCO.2015.65.9128 27138577

